# The complete mitochondrial genome of *Artemia salina* Leach, 1819 (Crustacea: Anostraca)

**DOI:** 10.1080/23802359.2021.1992315

**Published:** 2021-10-20

**Authors:** Gusang Deji, Chi Zhang, Liying Sui, Xuekai Han

**Affiliations:** a Tibet Academy of Agricultural and Animal Husbandry Sciences, Lhasa, PR China; b Institute of Fisheries Science, Tibet Academy of Agricultural and Animal Husbandry Sciences, Lhasa, PR China; c Asian Regional Artemia Reference Center, Tianjin University of Science and Technology, Tianjin, China

**Keywords:** *Artemia salina*, mitochondrial genome, phylogenetic

## Abstract

In the study, the complete mitochondrial genome of *Artemia salina* was reported for the first time. The mitochondrial genome of *A. salina* is 15,762 bp in length, with the typical structure of 13 protein-coding genes (PCGs), 22 transfer RNA genes (tRNAs), and two ribosomal RNA genes, and a major non-coding region (CR). Phylogenetic analysis showed that *A. salina* has a much closer relationship with *A. persimilis* compared to other *Artemia* species. The complete cp genome sequence of *A. salina* reported here provided an essential resource for further population genetics research and germplasm conservation on *Artemia*.

Brine shrimp *Artemia* (Arthropoda, Crustacea, Anostraca) inhabits hypersaline environments such as salt lakes and solar saltworks, which plays a biological regulatory role in salt field ecosystem. The genus *Artemia* is generally considered to contain seven bisexual species as well as some parthenogenetic *Artemia* populations with different polyploidy types (Asem et al. 2010). At present, the complete mitochondrial genome has been reported in four bisexual species, including *Artemia franciscana*, *Artemia urmiana*, *Artemia tibetiana*, and *Artemia sinica* (Valverde et al. 1994; Zhang et al. 2013; Asem et al. 2019). As for *Artemia salina*, a bisexual species endemic to the Mediterranean Basinare, is being threatened by habitat loss and the ongoing *A. franciscana* invasion (Muñoz et al. 2008). However, its complete mitochondrial genome has not been reported and characterized. Herein, we characterized the complete mitochondrial genome sequence of *A. salina* using Illumina sequencing, and a phylogenetic analysis was performed to investigate the phylogenetic relationships of *A. salina* with other *Artemia* species.

The cysts of *A. salina* were collected from Sebkha Eladhibet Saltworks, Tunisia (**latitude** 11.4342 and **longitude** 33.1060). The specimen was deposited at the Asian Regional Artemia Reference Center (Tianjin University of Science and Technology, Tianjin, China) (Liying Sui, suily@tust.edu.cn) under the voucher number 1632. The genomic DNA was extracted form cysts using TIANGEN^®^TIANamp Genomic DNA Kit (Tianjin, China). Then, the high quality gDNA was sequenced using Illumina Novaseq6000 platform with 350 bp insert size. The complete mitochondrial genome was assembled using SPAdes v.3.5.0 (http://cab.spbu.ru/software/spades/) with *A. franciscana* (GenBank accession number: X69067) as reference. The genome was first annotated with MITOS (http://mitos.bioinf.uni-leipzig.de/index.py) and ORF Finder (https://www.ncbi.nlm.nih.gov/orffinder/), then the reference mitochondrial map and BLAST (https://blast.ncbi.nlm.nih.gov/Blast.cgi) were used to verify the accuracy of the results and make corrections. The tRNA genes were predicted using the ARWEN (http://mbio-serv2.mbioekol.lu.se/ARWEN/) and tRNAscan-SE 2.0 (http://lowelab.ucsc.edu/tRNAscan-SE/) online software.

The complete mitochondrial genome of *A. salina* (GenBank accession number: MZ199177) is 15,762 bp in length, with the typical structure of 13 protein-coding genes (PCGs), 22 transfer RNA genes (tRNAs), and two ribosomal RNA genes, and a major non-coding region (CR) called the D-loop. The length variations are mainly confined to the D-loop region. The base composition is 30.80% A, 17.49% C, 17.98% G, and 33.73% T, with an A + T content of 64.53%. Just five PCGs (*cox1*, *atp6*, *cox3*, *cytb*, and *nd1*) began with the common ATG start codon. Stop codons included TAA (*cox3*, *nd2*, *atp8*, *atp6*, *nd3*, and *cytb*) and TAG (*nd1*, *nd4l*, and *nd6*). There are four genes ended with the incomplete stop codon T (*cox1*, *cox2*, *nd4*, and *nd5*). The 12S rRNA and16S rRNA were separated by the *trnV*. The length of *rrnL* and *rrnS* is 1144 bp and 711 bp, respectively. The 22 tRNA genes size varies from 59 to 67 bp, respectively.

To further explore the phylogenetic relationships among *Artemia* species, the complete mitochondrial genome sequences of five bisexual *Artemia* species were downloaded from GenBank, and the software MEGA 7.0 was used to make phylogenetic analysis by maximum-likelihood (ML) method with the Kimura 2-parameter model (Kumar et al. 2016). The results showed that *A. salina* at the base position of *Artemia* in this tree, and *A. salina* has a much closer relationship with *A. persimilis* compared to other *Artemia* species (Figure 1). The mitochondrial genome sequence of *A. salina* reported here can provide important information for population genetics and evolutionary studies of *Artemia*.

**Figure 1.**
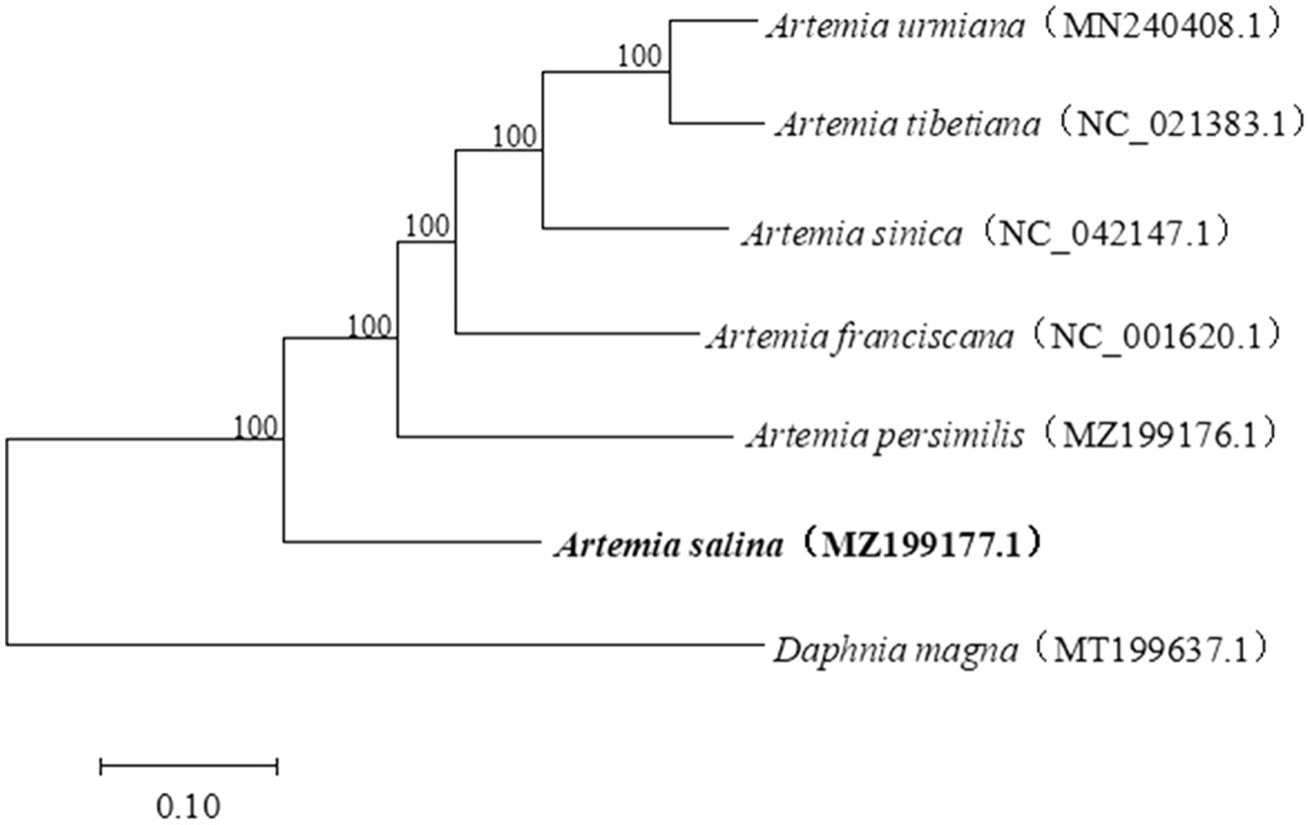
Phylogenetic tree showing the relationship among *A. sinica* and other species from the *Artemia*. The numbers on each node are the bootstrap support values. *Daphnia magna* was selected as an outgroup.

## Data Availability

The genome sequence data that support the findings of this study are openly available in GenBank of NCBI at https://www.ncbi.nlm.nih.gov under the accession number MZ199177. The associated BioProject, SRA, and BioSample numbers are PRJNA749918, SRR15292986, and SAMN20425675, respectively.
